# Global Warming and Type 2 Diabetes: Sex and Gender as Drivers in a Multidimensional Feedback Loop

**DOI:** 10.1002/gch2.70140

**Published:** 2026-10-18

**Authors:** Massimo D'Archivio, Giulia Zanchi, Alessia Tammaro, Roberta Masella, Claudia Cataldo

**Affiliations:** ^1^ Gender‐Specific Prevention and Health Unit Centre For Gender‐Specific Medicine Istituto Superiore di Sanità Rome Italy

**Keywords:** climate change, dietary behaviors, gender, global warming, metabolic health, type 2 diabetes

## Abstract

Climate change is increasingly recognized as a determinant of metabolic health. Emerging evidence indicates that rising temperatures impair glucose homeostasis—specifically through reduced brown adipose tissue activity and increased insulin resistance—thereby exacerbating type 2 diabetes mellitus (T2DM) risk. Concurrently, food systems drive substantial greenhouse gas emissions (GHGE), with gender‐differentiated dietary patterns uniquely influencing both environmental impact and health outcomes. In this perspective, we move beyond well‐established separate links to propose an original integrated and systemic conceptual framework. This framework connects gender‐related drivers in dietary behaviors, GHGE, global warming, brown adipose tissue dysfunction, and T2DM risk into an integrated multidimensional feedback model, providing a novel basis to generate hypotheses for future mechanistic, epidemiological, and intervention studies, supporting gender‐sensitive prevention strategies.

## Abstract 2

This graphical abstract illustrates how gender‐based dietary choices impact climate change and health. Higher plant consumption (associated with females) leads to climate‐friendly outcomes. Conversely, higher meat consumption (associated with males) drives climate change. This environmental shift makes Brown Adipose Tissue (BAT) less active, resulting in a weaker effect on glucose regulation and an increased risk of Type 2 Diabetes.

**Abbreviations**
BAT: brown adipose tissue
GDM: gestational diabetes mellitus
GHGE: greenhouse gas emissions
T2DM: type 2 diabetes

## 1 Introduction

Climate change is widely recognized as the most urgent and complex challenge of the 21st century, with profound, multifaceted effects on both the environment and human health. Over recent decades, scientific attention has increasingly focused on the intricate links among dietary choices, environmental sustainability, and public health. It is now evident that individual consumption patterns not only impact personal well‐being but also influence planetary health and the progression of chronic diseases such as type 2 diabetes (T2DM). Unhealthy diets, particularly those rich in red meat and ultra‐processed foods, disproportionately contribute to global warming. The livestock sector plays an important role in climate change, with emissions estimated at 7.1 gigatonnes CO_2_‐eq per annum, representing 14.5 percent of human‐induced greenhouse gas emissions (GHGE) [1]. In this framework, disproportionate dietary habits affect every stage of the food supply chain, including agriculture, land use, and deforestation for grazing and fodder production, further elevating CO_2_ emissions [2].

This environmental pressure is further confirmed by recent data from the updated report of the EAT‐Lancet Commission, which underscores that food systems remain responsible for approximately one‐third of global anthropogenic GHGE, asserting that a global dietary transformation toward plant‐forward patterns could reduce agricultural land use and food‐related GHGE by up to 50% by 2050 [3].

Metabolic diseases are increasingly recognized as critical outcomes within the expanding body of evidence linking climate change and human health. Among these, T2DM occupies a central position because of its high prevalence, chronic course, and sensitivity to thermal stress. Exposure to extreme temperatures is associated with higher diabetes‐related risk, increased hospitalizations and emergency visits, and greater mortality, suggesting that heat and cold waves can influence outcomes in people with diabetes as well as the risk of dysglycemia in the general population [4, 5].

Concerning underlying biological mechanisms, heat stress may impair glucose homeostasis through reduced thermoregulatory capacity, altered insulin sensitivity, inflammation, oxidative stress, and dysfunction of brown adipose tissue (BAT) [6]. BAT is a metabolically active organ involved in thermogenesis and glucose handling; heat stress can reduce BAT activity and worsen mitochondrial integrity in diabetic models [7]. These findings support the plausibility of a mechanistic link between climate warming and metabolic deterioration. However, the climate‐diabetes relationship is multidimensional. Indeed, dietary choices act as an upstream driver that both directly influences GHGE and indirectly affect T2DM risk. Within this complex multidimensional context, accumulating evidence underscores the significant influence of sex and gender on dietary behaviors and related health outcomes [8, 9]. These differences can enhance understanding and education in food and nutrition sciences [10, 11]. Sex‐ and gender‐related disparities arise from interaction among biological, cultural, and socioeconomic factors. For example, women, on average, show greater awareness of environmental issues and a stronger propensity toward sustainable eating habits [12, 13]. Additionally, biological sex factors, particularly hormonal changes during pregnancy and menopause, interact with heat exposure, making women particularly vulnerable to climate‐related health risks [14]. A recent retrospective cohort study has significantly strengthened this evidence, demonstrating that early pregnancy warm‐season exposure significantly amplifies the risk of adverse maternal metabolic outcomes, including gestational diabetes mellitus (GDM) [15]. Hormonal fluctuations during these key life stages can indeed modify metabolic responses to environmental changes. These findings highlight the need to integrate sex‐ and gender‐ specific perspectives in public health strategies to better understand climate adaptation under stressful conditions.

While the epidemiological associations and biological pathways consist of data already established in literature, the specific originality of this perspective analysis lies in the formulation of an original, overarching multidimensional feedback framework. Previous works have treated the dietary GHGE footprint and temperature‐induced metabolic impairments as separate parallel phenomena. Here, we perform a novel integrative‐systemic conceptualization, showing that gender‐differentiated dietary choices do not merely represent localized preferences, but act as an upstream behavioral driver that structurally modulates downstream physiological vulnerability affecting climate‐warming effects: gender‐related dietary choices influence GHGE, GHGE contribute to global warming, and global warming may worsen glucose homeostasis and T2DM risk through heat‐related metabolic stress, shape the multidimensional feedback loop.

## 2 Climate Change and Its Impact on Diabetes

### 2.1 T2DM Epidemiology and Climate Change at Global Scale

Worldwide, approximately 537 million adults aged 20 to 79 live with T2DM, a number expected to increase to 643 million by 2030 and 783 million by 2045. Notably, over three‐quarters of these individuals reside in low‐ and middle‐income countries, highlighting significant global health disparities [16, 17]. This expected rise in diabetes prevalence underscores the urgent need for coordinated global action to address both the environmental and social determinants of metabolic health. T2DM is associated with severe complications, that substantially increase morbidity and mortality and severely impair patients' quality of life [18, 19]. The cumulative impact of these complications not only affects individuals but also places a significant strain on families, communities, and healthcare systems. The economic impact is profound: the estimated global direct health cost on diabetes in 2019 was USD 760 billion, projected to grow to USD 825 billion by 2030 and USD 845 billion by 2045 [20], placing a heavy financial burden on healthcare systems, especially in resource‐limited settings [21].

A comprehensive epidemiological study linking outdoor temperature to T2DM incidence [22] demonstrated that rising ambient temperatures are associated with an increase in diabetes incidence. Specifically, for every 1°C increase, the incidence of diabetes in the United States rises by 0.314 per 1000 people, and the global prevalence of glucose intolerance increases by 0.170% [22]. This suggests a strong link between higher temperatures and increased diabetes risk. Higher temperatures impair insulin receptor signaling, reduce BAT thermogenesis, and induce systemic inflammation and oxidative stress, all contributing to insulin resistance and impaired glucose metabolism [23, 24]. This metabolic pathway is further corroborated by very recent functional data confirming that elevated ambient temperatures significantly decrease cellular fat storage through specific modifications of insulin receptor signaling, highlighting a direct biological linkage between ambient heat and cellular metabolic homeostatic failure [25].

### 2.2 Mechanistic Implication of Climate Change on Metabolic Health

The rising of global temperatures and the increased frequency of heat waves pose significant health risks, including impacts on metabolic health [22, 26, 27]. Prolonged exposure to heat can overwhelm the body's thermoregulatory systems, leading to heat‐related illnesses such as heat exhaustion and heat stroke [28, 29], conditions characterized by the body's inability to maintain its core temperature around the normal range (37°C or 98.6°F). Heat stress disrupts physiological processes like metabolism, vasodilation, and sweating, which can contribute to insulin resistance and the development of T2DM and GDM [30, 31, 32]. In particular, heat stress reduces insulin sensitivity, exacerbating metabolic dysfunction [33]. These physiological disruptions may be further aggravated by dehydration, electrolyte imbalances, and changes in physical activity patterns during periods of extreme heat. BAT plays a crucial role in thermogenesis, which is the process of heat production in living organisms [34]. Thermogenesis is primarily achieved through the activation of uncoupling protein 1 (UCP1) in mitochondria, which allows the conversion of chemical energy into heat [35]. BAT helps maintain glucose homeostasis and improve insulin sensitivity by burning lipids and glucose to produce heat. BAT activity inversely correlates with ambient temperature: cold exposure activates it, boosting glucose uptake, while elevated temperatures suppress this beneficial function, worsening glucose regulation, and increasing diabetes risk [36]. Strategies to activate or preserve BAT may offer novel approaches to improve glucose control and lower diabetes risk in the context of rising global temperatures [37]. This thermogenic role of BAT is crucial in preventing obesity and related metabolic disorders, like T2DM [38]. A very recent study investigated the effect of short‐term heat stress on T2DM mice, focusing on the role of BAT [7]. The results showed that heat stress significantly aggravated metabolic disorders in T2DM mice, leading to a marked increase in fasting blood glucose and insulin resistance, along with worsened dyslipidemia as demonstrated by increased triglycerides and LDL, and decreased HDL levels. Mechanistically, heat stress was found to inhibit BAT activity by significantly reducing the mRNA expression of the thermogenic gene UCP1 and decreasing BAT glucose uptake. The failure of BAT to clear glucose and regulate metabolism contributes directly to the observed hyperglycemia and insulin resistance. Functional inhibition of BAT is directly linked to structural damage in adipocyte mitochondria. This damage impairs oxidative phosphorylation and thermogenesis, worsening metabolic dysregulation. The authors of this study clearly provide mechanistic support to link temperature and diabetes, indicating that heat stress aggravates the dysregulation of glucose and lipid metabolism, exacerbates mitochondrial dysfunction in BAT, and reduces the activity of BAT in T2DM mice [7]. On the other hand, it has been demonstrated that cold exposure can improve insulin sensitivity and reduce fasting glycaemia in individuals with T2DM. A landmark study by Hanssen et al. [36] showed that exposing T2DM patients to moderate cold (14‐15°C) for 10 days significantly improved insulin sensitivity without weight changes, likely through increased fatty acid flux to BAT and increased glucose uptake by skeletal muscle and liver, the primary insulin target tissues [36]. BAT activity is inversely correlated with outdoor temperature, being higher in winter [39]. Conversely, elevated temperatures reduce BAT activity, potentially worsening glucose regulation, and increasing diabetes risk [27]. Rising global temperatures may thus contribute to the T2DM epidemic by suppressing BAT function [40]. Supporting this hypothesis, Lee P [23]. found a positive association between outdoor temperature and glycated hemoglobin (HbA1c) levels, indicating environmental temperature influences systemic glucose homeostasis.

### 2.3 Sex‐Specific Outcomes in T2DM‐Temperature Relationship

Temperature fluctuations also affect GDM risk. Seasonal trends show a higher prevalence of GDM during warmer months [41]. Teyton et al. identified critical windows in the second trimester [42] during which a 10°C increase in temperature corresponds to a 6–9% rise in GDM risk [43]. Similar seasonal and temperature‐related increases in GDM incidence have been reported by other studies [44, 45]. These findings underscore the importance of monitoring environmental conditions during pregnancy and adopting mitigation measures to protect maternal and fetal health.

Higher daily average temperatures are also associated with increased diabetes‐related hospitalizations, especially among older adults [5]. Conversely, lower temperatures, younger age, female sex, and non‐diabetic status are linked to higher BAT prevalence [46, 47]. In summary, the evidence consistently demonstrates a link between rising ambient temperatures and an increased incidence and prevalence of T2DM. Understanding these temperature‐related mechanisms is essential for developing effective prevention and management strategies to address the growing global diabetes burden.

## 3 Gender Drivers and Their Impact on Climate Change

Recent research highlights the significant role of the food sector in driving climate change, with about one‐third of global GHGE originating from food systems [48]. Among all food sources, meat—especially from ruminant animals—is a major contributor with approximately 14% of global GHGE [49]. As a result, dietary guidelines increasingly recommend shifting toward plant‐based protein sources to promote environmental sustainability [50, 51]. A global move toward low‐meat diets could cut the economic costs of climate change mitigation by up to 50% by 2050 [52]. The EAT–Lancet Commission's “planetary health diet” emphasizes plant‐based foods and limited animal products, and its adoption could reduce global dietary emissions by approximately 17% and prevent millions of diet‐related deaths annually [3, 53]. The adoption of these diets requires coordinated efforts from governments, industry, and civil society to overcome barriers related to food preferences, cultural traditions, and economic constraints.

Western diets, characterized by high consumption of red meat and dairy, contribute disproportionately to GHGE and resource depletion [54]. The environmental impact of food choices varies widely, making it crucial to collect dietary data at the individual level to design targeted and effective policies. Importantly, nutritional behaviors differ significantly across demographic groups, including distinctions based on sex and gender. Gender is a key driver shaping food choices, influencing not only what people eat but also their motivations and attitudes. Research consistently shows that meat consumption is often linked to traditional masculinity [55]. It is well known that women tend to express greater concern for animal welfare and the environment, which may explain their higher rates of vegetarianism and veganism [3]. Studies also reveal that women generally have better awareness of climate change and are more concerned about its impacts than men [56]. These gender differences in diet are shaped by cultural norms, education, socioeconomic status, and personal beliefs [55, 56, 57]. Furthermore, socialization from an early age reinforces these patterns, exposing boys and girls to different messages about food, health, and environmental responsibility. Media and marketing further perpetuate gendered stereotypes about diet, influencing habits throughout life [58, 59]. A recent cross‐cultural study provides robust evidence that women generally eat less meat than men, with this difference becoming more pronounced in highly developed countries with greater gender equality [60]. This suggests that as society's progress and gender equality improves, differences in dietary habits—especially meat consumption—may become more marked rather than diminish, reflecting the complex relationship between social development and individual food choices [61]. Auclair et al. [51] investigates the effects of partially replacing animal protein foods with plant protein foods in Canadian consumers, assessing on nutritional, health, and climate outcomes. The authors found that replacing 50% of red and processed meat with plant‐based proteins could reduce GHGE by up to 25% of kg CO_2_‐equivalent per person per day, with men seeing a greater reduction than women, likely reflecting higher baseline meat consumption (27% for males, 21% for females; *p* < 0.0001). However, when comparing climate footprint across demographic groups, baseline data show higher diet‐related GHGE in men than in women (5.85 vs. 3.88 kg CO_2_ eq/day,) [62]. These absolute values can be misleading unless normalized for total caloric intake. In this regard, a notable study by Hallstrom E. et al. [63] found that although men had higher overall diet‐related GHGE (about 36% higher), normalizing the results per 1000 kcal eliminate the difference, suggesting that the greater diet‐related GHGE in men was mainly due to higher total energy intake rather than intrinsically different food choices. Conversely, other studies [64, 65, 66] have reported that men still exhibit a higher dietary climate impact even for the same amount of calories. Taken together, these findings support the view that men tend to consume a larger proportion of carbon‐intensive ruminant meat, while women have a relative higher intake of plant‐based foods and greater adherence to sustainable dietary guidelines.

Switching to plant‐based diets, such as vegan or Mediterranean diets, can significantly reduce GHGE compared to typical Western diets: reductions have been estimated at up to 50% (kg CO_2_ equivalent per year per functional unit per year) for vegan diet and 20–30% for Mediterranean diets [67]. Here, the term Functional Unit refers to a standardized measure that captures the basic functions of food intake (primarily adequate nutrition and satiety), enabling fair comparison of environmental impacts by accounting for nutritional equivalence rather than food mass alone. Among dietary patterns, vegan diets exhibits the lowest environmental impact, and omnivorous diets the highest. Allenden et al. [68] assigned environmental impact scores from 1 to 10 to six dietary patterns (omnivore, Mediterranean, pescatarian, flexitarian/semi‐vegetarian, vegetarian, and vegan), where higher scores indicate greater environmental benefit. The omnivore diet received the lowest score (1), the vegan diet highest (10), and the pescatarian diet ranked relatively among intermediates (6.81).

In summary, sex and gender differences in dietary choices are a major determinant of the environmental impact of food systems. Male dietary patterns may also indirectly increase metabolic risk by elevating exposure to climate‐related harms. These findings underscore the importance of incorporating sex and gender considerations into public health interventions aimed at both climate‐change mitigation and diabetes prevention.

## 4 Discussion on Multidimensional Feedback Loop and Future Perspective

Our perspective work summarizes key drivers and outcomes also by sex and gender lens which interplay in the climate‐diet‐T2DM nexus (Table 1), and proposes an integrated‐systemic, testable model linking gendered dietary behaviors, food‐system GHGE, climate warming, BAT dysfunction, and increased T2DM risk. Its primary novelty is framing sex‐ and gender‐related food choices as upstream, modifiable determinants that shape environmental exposures and thereby modulate biological vulnerability to heat‐induced metabolic dysfunction. While prior work has described individual links among diet, GHGE, warming, and metabolic disease, we synthesize them into a single multidimensional feedback loop that generates specific hypotheses for mechanistic, eco‐epidemiological, and intervention research as visualized in Figure 1.

**TABLE 1:** Key quantitative data derive from the cited references in the text, on epidemiological, modeling, and estimation of carbon footprint studies among multiple sources, summarizing the multidimensional feedback loop of temperature‐diabetes relationships and its implications with the sex‐gender‐specific drivers and outcomes included in the loop.

Parameter	Value	*Reference*
Anthropogenic GHG emissions from livestock	^*^14.5%	*Gerber* et al. *(2013)*
Diet‐related GHGE emissions men vs. women	3.88 vs. 3.03 (kg CO_2_eq/1000 kcal/day)	*Hiort* et al. (2020)
Diet‐related GHGE emissions men vs. women	1031 vs 777 (kg CO_2_eq/megajoules/day)	*Hyland* et al. (2017)
Diet‐related GHGE emissions men vs. women (%)	Low GHGE diet: 43.9 *vs* 56.1 High GHGE diet: 56.5 *vs* 43.5 (kg CO_2_eq/1000 kcal/day)	*Rose* et al. (2019)
GHG reduction with 50% red/processed meat substitution	Up to 25% kg CO_2_ eq/person/day (27% men, 21% women; P<0.0001)	*Auclair* et al. *(2024)*
US diabetes incidence per +1°C of Environmental temperature	+0.314/1,000 people	*Blauw* et al. *(2017)*
Global glucose intolerance prevalence per +1°C of Environmental temperature	+0.170	*Blauw* et al. *(2017)*
Rise in GDM in the second trimester per +10°C of Environmental temperature	+6–9% in risk and incidence	*Booth* et al. *(2017); Teyton* et al. *(2023); Zhang* et al. *(2021)*
Environmental impact scores of diets (1–10 scale)	Omnivore: 1; Vegan: 10; Pescatarian: 6.81	*Allenden* et al. *(2022)*

* of human‐induced greenhouse gas emissions.

**FIGURE 1:**
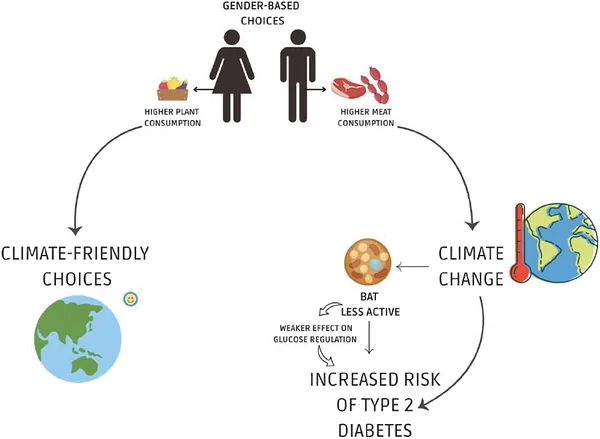
Feedback loop among Gender‐Specific Dietary Choices, greenhouse gas emissions (GHGE), Climate Change (CC), Brown adipose tissue (BAT) and type 2 diabetes (T2DM).

The model highlights four interrelated points. First, gendered food‐choice patterns—particularly higher ruminant‐meat consumption in men and relatively greater plant‐based intake in women—can meaningfully influence population‐level GHGE and thus contribute to global warming. Second, rising temperatures plausibly impair glucose homeostasis by suppressing BAT activity, disrupting insulin signaling, and promoting inflammation and mitochondrial dysfunction; these effects interact with sex‐specific life‐course factors (e.g., pregnancy, menopause) that alter metabolic resilience. Third, the processes form a reinforcing loop: gendered diets increase GHGE, GHGE drive global warming, global warming worsens metabolic outcomes, and a growing disease burden amplifies societal vulnerability. Fourth, because gendered dietary patterns occupy a proximal position in this loop, they represent a high‐leverage target for interventions with co‐benefits for climate mitigation and metabolic health.

From this framework, we derive testable predictions and research priorities. Mechanistic studies should quantify how acute and chronic heat exposure affects BAT, insulin signalling, and mitochondrial function, stratified by sex and reproductive stage. Longitudinal eco‐epidemiological studies should integrate high‐resolution temperature exposure, sex‐disaggregated dietary data, biomarkers of BAT activity, and incident dysglycaemia. Intervention trials should evaluate gender‐tailored dietary strategies (for example, reducing ruminant meat intake among men rather than focusing solely on differences in caloric requirements) combined with environmental measures (cooling, occupational protections), assessing both GHGE and metabolic endpoints. Integrated modeling is needed to estimate population‐level impacts, cost‐effectiveness, and equity consequences of combined dietary and climate policies.

Policy implications flow directly from the model: mitigation strategies will be more effective if they explicitly incorporate sex and gender, pairing promotion of plant‐forward diets with measures to reduce heat exposure and protect vulnerable subgroups (notably pregnant and perimenopausal women and those with existing metabolic disease). National surveillance should collect sex‐ and gender‐disaggregated indicators on diet, thermal exposure, and diabetes outcomes to inform targeted action.

By positioning sex and gender as central, modifiable drivers in the climate–diet–metabolic nexus, this perspective offers a concise conceptual roadmap to align climate mitigation with diabetes prevention. Interdisciplinary research and coordinated, gender‐sensitive policy are required to test the model and translate its insights into effective, equitable interventions.

## Funding

The research leading to these results has received funding from the Italian National Institute of Health (ISS) “Bando Ricerca Indipendente 2023”—A combined approach based on a medical nutrition intervention and pharmacological treatment can affect the inflammatory status and liver function in men and women with metabolic syndrome and type 2 diabetes mellitus Project code: ISS20‐3336764b7f71.

## Conflicts of Interest

The authors declare no conflicts of interest.

## Data Availability

The contributions presented in the study are included in the article. Further inquiries can be directed to the corresponding author.
